# Supplementary material for: Brain-wide arousal signals are segregated from movement planning in the superior colliculus of the macaque
Source: eLife. 2026 Jun 8;13:RP99278. doi: 10.7554/eLife.99278 (PMC13246003; doi:10.7554/eLife.99278)
Supplement: Peer review history

# Editor report: eLife Assessment

**DOI:** 10.7554/eLife.99278.4.sa0

**Authors:** Terrence R Stanford

**Affiliations:** Wake Forest University School of Medicine United States

**Keywords:** Solid, Valuable

This study presents a **valuable** finding relating to how the state of arousal is represented within the superior colliculus (SC), a principal visuo-oculomotor structure. The main conclusion that the SC's neural representation of arousal is segregated from motor related output appears to have **solid** support by the data. The work will be of interest to sensory, motor and cognitive neuroscientists.

# Referee report: Reviewer #1 (Public review)

**DOI:** 10.7554/eLife.99278.4.sa1

Summary:

Johnston and Smith used linear electrode arrays to record from small populations of neurons in the superior colliculus (SC) of monkeys performing a memory-guided saccade (MGS) task. Dimensionality reduction (PCA) was used to reveal low-dimensional subspaces of population activity reflecting the slow drift of neuronal signals during the delay period across a recording session (similar to what they reported for parts of cortex: Cowley et al., 2020). This SC drift was correlated with a similar slow-drift subspace recorded from the prefrontal cortex, and both slow-drift subspaces tended to be associated with changes in arousal (pupil size). These relationships were driven primarily by neurons in superficial layers of the SC, where saccade sensitivity/selectivity is typically reduced. Accordingly, delay-period modulations of both spiking activity and pupil size were independent of saccade-related activity, which was most prevalent in deeper layers of the SC. The authors suggest that these findings provide evidence of a separation of arousal- and motor-related signals. The analysis techniques expand upon the group's previous work and provides useful insight into the power of large-scale neural recordings paired with dimensionality reduction. This is particularly important with the advent of recording technologies which allow for the measurement of spiking activity across hundreds of neurons simultaneously. Together, these results provide a useful framework for comparing how different populations encode signals related to cognition, arousal, and motor output in potentially different subspaces.

Comments on revised manuscript:

The authors have done a very good job of responding to all of the reviewers' concerns.

# Referee report: Reviewer #2 (Public review)

**DOI:** 10.7554/eLife.99278.4.sa2

Summary:

Neurons in motor-related areas have increasingly shown to carry also other, non-motoric signals. This creates a problem of avoidance of interference between the motor and non-motor-related signals. This is a significant problem that likely affects many brain areas. The specific example studied here is interference between saccade-related activity and slow-changing arousal signals in the superior colliculus. The authors identify neuronal activity related to saccades and arousal. Identifying saccade-related activity is straightforward, but arousal-related activity is harder to identify. The authors first identify a potential neuronal correlate of arousal using PCA to identifying a component in the population activity corresponding to slow drift over the recording session. Next, they link this component to arousal by showing that the component is present across different brain areas (SC and PFC), and that it is correlated with pupil size, an external marker of arousal. Having identified an arousal-related component in SC, the authors show next that SC neurons with strong motor-related activity are less strongly affected by this arousal component (both SC and PFC). Lastly, they show that SC population activity pattern related to saccades and pupil size form orthogonal subspaces in the SC population.

Strengths:

A great strength of this research is the clear description of the problem, its relationship with the performed analysis and the interpretation of the results. The paper is very well written and easy to follow.

An additional strength is the use of fairly sophisticated analysis using population activity.

Weaknesses:

(1) The greatest weakness in the present research is the fact that arousal is a functionally less important non-motoric variable. The authors themself introduce the problem with a discussion of attention, which is without any doubt the most important cognitive process that needs to be functionally isolated from oculomotor processes. Given this introduction, one cannot help but wonder, why the authors did not design an experiment, in which spatial attention and oculomotor control are differentiated. Absent such an experiment, the authors should spend more time on explaining the importance of arousal and how it could interfere with oculomotor behavior.

(2) In this context, it is particularly puzzling that one actually would expect effects of arousal on oculomotor behavior. Specifically, saccade reaction time, accuracy, and speed could be influenced by arousal. The authors should include an analysis of such effects. They should also discuss the absence or presence of such effects and how they affect their other results.

(3) The authors use the analysis shown in Figure 6D to argue that across recording sessions the activity components capturing variance in pupil size and saccade tuning are uncorrelated. however, the distribution (green) seems to be non-uniform with a peak at very low and very high correlation, specifically. The authors should test if such an interpretation is correct. If yes, where are the low and high correlations respectively? Are there potentially two functional areas in SC?

Comments on the first revision:

My main concern with the paper is really two-fold. First, I think it is only incremental and adds next to no useful information about the SC. That might not be a fair criticism and certainly is purely subjective, but it affects the standards that eLife has on significance thresholds for papers. As such, this is an issue the editors should talk about.

Second, my main concern with the substance of the paper is that the authors jump immediately into an analysis of the 'arousal-related' effects on SC activity. Before that, I would like to see some behavioral indicators of arousal, such as RT differences, pupil size (the talk about this), or accuracy. The authors first need to describe the objective behavioral indicators of the level of arousal. Using these indices, they need to establish that there are meaningful differences in the level of arousal across the recording session. Having done so, they can proceed to link changes in SC activity with levels of arousal.

Instead, in its current form, the authors find changes in SC activity and describe them immediately as 'arousal-related'. I hope it is clear why that is premature. The 'slow-drift' fluctuations are presumed to be related to arousal, but they could be meaningless random fluctuations, or related to some other cognitive process.

Other than this conceptual issue, I do not have major problems with the analysis per se.

Comments on the latest version:

They have constructively responded to my concerns. I think 'incomplete' should be replaced with 'solidly supported'.

# Referee report: Reviewer #3 (Public review)

**DOI:** 10.7554/eLife.99278.4.sa3

Summary:

This study looked at slow changes in neuronal activity (on the order of minutes to hours) in the superior colliculus (SC) and prefrontal cortex (PFC) of two monkeys. They found that SC activity shows slow drift in neuronal activity like in the cortex. They then computed a motor index in SC neurons. By definition, this index is low if the neuron has stronger visual responses than motor response, and it is high if the neuron has weaker visual responses and stronger motor responses. The authors found that the slow drift in neuronal activity was more prevalent in the low motor index SC neurons and less prevalent in the high motor index neurons. In addition, the authors measured pupil diameter and found it to correlate with slow drifts in neuronal activity, but only in the neurons with lower motor index of the SC. They concluded that arousal signals affecting slow drifts in neuronal modulations are brain-wide. They also concluded that these signals are not present in the deepest SC layers, and they interpreted this to mean that this minimizes the impact of arousal on unwanted eye movements.

Strengths:

The paper is clear and well-written.

Showing slow drifts in the SC activity is important to demonstrate that cortical slow drifts could be brain-wide.

Weaknesses:

The authors find that the SC cells with the low motor index are modulated by pupil diameter. However, this could be independent of an "arousal signal". These cells have substantial visual sensitivity. If the pupil diameter changes, then their activity should be influenced since the monkey is watching a luminous display. So, in this regard, the fact that they do not see "an arousal signal" in the most motor neurons (through the pupil diameter analyses) is not evidence that the arousal signal is filtered out from the motor neurons. It could simply be that these neurons simply do not get affected by the pupil diameter because they do not have visual sensitivity.

Comments on revisions:

The authors have given due consideration to the possibility that SC signaling of arousal could be at least in part due to changes in pupil size related responses to ambient light. Discussion of this point in the most recent revision helps to mitigate this concern.

# Author comment: Author response

**DOI:** 10.7554/eLife.99278.4.sa4

**Authors:** Richard Johnson, Matthew A Smith

**Affiliations:** University of Pittsburgh Pittsburgh United States; Carnegie Mellon University Pittsburgh United States

The following is the authors’ response to the previous reviews

**Public Reviews:**

**Reviewer #1 (Public review):**

Summary:

Johnston and Smith used linear electrode arrays to record from small populations of neurons in the superior colliculus (SC) of monkeys performing a memory-guided saccade (MGS) task. Dimensionality reduction (PCA) was used to reveal low-dimensional subspaces of population activity reflecting the slow drift of neuronal signals during the delay period across a recording session (similar to what they reported for parts of cortex: Cowley et al., 2020). This SC drift was correlated with a similar slow-drift subspace recorded from the prefrontal cortex, and both slow-drift subspaces tended to be associated with changes in arousal (pupil size). These relationships were driven primarily by neurons in superficial layers of the SC, where saccade sensitivity/selectivity is typically reduced. Accordingly, delay-period modulations of both spiking activity and pupil size were independent of saccade-related activity, which was most prevalent in deeper layers of the SC. The authors suggest that these findings provide evidence of a separation of arousal- and motor-related signals. The analysis techniques expand upon the group's previous work and provides useful insight into the power of large-scale neural recordings paired with dimensionality reduction. This is particularly important with the advent of recording technologies which allow for the measurement of spiking activity across hundreds of neurons simultaneously. Together, these results provide a useful framework for comparing how different populations encode signals related to cognition, arousal, and motor output in potentially different subspaces.

Comments on revised manuscript:

The authors have done a very good job of responding to all of the reviewers' concerns.

No weaknesses to address.

**Reviewer #2 (Public review):**

Weaknesses:

(1) The greatest weakness in the present research is the fact that arousal is a functionally less important non-motoric variable. The authors themself introduce the problem with a discussion of attention, which is without any doubt the most important cognitive process that needs to be functionally isolated from oculomotor processes. Given this introduction, one cannot help but wonder, why the authors did not design an experiment, in which spatial attention and oculomotor control are differentiated. Absent such an experiment, the authors should spend more time on explaining the importance of arousal and how it could interfere with oculomotor behavior.

(2) In this context, it is particularly puzzling that one actually would expect effects of arousal on oculomotor behavior. Specifically, saccade reaction time, accuracy, and speed could be influenced by arousal. The authors should include an analysis of such effects. They should also discuss the absence or presence of such effects and how they affect their other results.

(3) The authors use the analysis shown in Figure 6D to argue that across recording sessions the activity components capturing variance in pupil size and saccade tuning are uncorrelated. however, the distribution (green) seems to be non-uniform with a peak at very low and very high correlation specifically. The authors should test if such an interpretation is correct. If yes, where are the low and high correlations respectively? Are there potentially two functional areas in SC?

Comments on revised manuscript:

I remain somewhat concerned that the authors jump immediately into an analysis of the 'arousal-related' effects on SC activity. Before that, I would like to see a more detailed discussion justifying the use pupil size alone (i.e., w/o other indicators such as RT) as indicative of fluctuations in general arousal that are causal to concomitant changes in SC activity. Instead, in its current form, the authors find changes in SC activity and describe them immediately as 'arousal-related'.

Other than this conceptual issue, I do not have major problems with the analysis per se.

We agree with the reviewer that we may have advanced into discussing arousal-related effects in the previous version of the manuscript without providing a thorough explanation for why we think the slow drift axis is associated with changes in the monkey’s arousal levels. Arousal has been linked to the size of the pupil as well as movements of the eyes in numerous previous studies. We have made the following changes in the revised manuscript to address the reviewer’s concern:

(1) When first describing how the spiking responses of SC neurons fluctuate over the course of a recording session (Lines 130-132), we have used the phrase "slow fluctuations in the spiking responses" rather than "arousal-related fluctuations in the spiking responses". Then, when describing these effects in more detail (Lines 136-147), we have explained why we think these fluctuations may be related to arousal. The following text has been added in the revised manuscript for clarification:

“We found that this low-dimensional pattern of activity in the SC was also correlated with pupil size in the present study and with simultaneously recorded data in the prefrontal cortex (PFC), pointing to a link between this brain-wide fluctuation and changes in the monkeys’ arousal levels while performing the task.” (Lines 136-147)

(2) We have changed the subheading in Line 183 of the revised manuscript from "Arousal-related fluctuations are present in the SC and correlated with pupil size and fluctuations in PFC activity" to "Slow fluctuations in SC spiking activity are correlated with pupil size and PFC activity". Given that we have not yet explained the results linking these fluctuations to arousal at this stage of the manuscript, we believe that this revised title is more accurate and avoids jumping too quickly to arousal-related fluctuations without first explaining the link between SC slow drift, pupil size and PFC activity.

(3) We have provided additional justification for using pupil size and PFC activity to assess whether SC slow drift is associated with changes in the monkeys’ arousal levels. In a previous study, we computed an identical slow drift axis for spiking responses in visual cortex (V4) and PFC, and investigated how these low-dimensional neural activity patterns, which were themselves strongly correlated, were associated with various eye-related metrics (e.g., pupil size, microsaccade rate, reaction time, saccade velocity). Results showed that pupil size was the strongest predictor of slow drift in V4 and PFC. Given that the eye metrics were also strongly correlated with each other, we believe that the observed relationship between SC slow drift, pupil size and PFC activity provides sufficient evidence to suggest that the fluctuations observed in the SC are arousal-related. The following text has been added to the Results section of the revised manuscript:

“Moreover, previous work in our laboratory computed a similar slow-drift axis using spiking activity in visual cortex (V4) and PFC, and investigated the relationship between these low-dimensional neural activity patterns and different eye-related metrics (e.g., pupil size, microsaccade rate, reaction time, saccade velocity). In addition to observing a strong correlation between V4 and PFC slow drift, we found that, relative to the other eye-related metrics, pupil size was the strongest predictor of these fluctuations (Johnston et al., 2022a). Thus, to further confirm the link between the SC slow drift axis and changes in the monkeys’ arousal levels while they performed the MGS task, we next sought to explore if projections onto the SC slow drift axis were associated with pupil size.” (Lines 236-344)

**Reviewer #3 (Public review):**

Summary:

This study looked at slow changes in neuronal activity (on the order of minutes to hours) in the superior colliculus (SC) and prefrontal cortex (PFC) of two monkeys. They found that SC activity shows slow drift in neuronal activity like in the cortex. They then computed a motor index in SC neurons. By definition, this index is low if the neuron has stronger visual responses than motor response, and it is low if the neuron has weaker visual responses and stronger motor responses. The authors found that the slow drift in neuronal activity was more prevalent in the low motor index SC neurons and less prevalent in the high motor index neurons. In addition, the authors measured pupil diameter and found it to correlate with slow drifts in neuronal activity, but only in the neurons with lower motor index of the SC. They concluded that arousal signals affecting slow drifts in neuronal modulations are brain-wide. They also concluded that these signals are not present in the deepest SC layers, and they interpreted this to mean that this minimizes the impact of arousal on unwanted eye movements.

Strengths:

The paper is clear and well-written.

Showing slow drifts in the SC activity is important to demonstrate that cortical slow drifts could be brain-wide.

Weaknesses:

The authors find that the SC cells with the low motor index are modulated by pupil diameter. However, this could be completely independent of an "arousal signal". These cells have substantial visual sensitivity. If the pupil diameter changes, then their activity should be influenced since the monkey is watching a luminous display. So, in this regard, the fact that they do not see "an arousal signal" in the most motor neurons (through the pupil diameter analyses) is not evidence that the arousal signal is filtered out from the motor neurons. It could simply be that these neurons simply do not get affected by the pupil diameter because they do not have visual sensitivity. So, even with the pupil data, it is still a bit tricky for me to interpret that arousal signals are excluded from the "output layers" of the SC.

Of course, the general conclusion is that the motor neurons will not have the arousal signal. It's just the interpretation that is different in the sense that the lack of the arousal signal is due to a lack of visual sensitivity in the motor neurons.

I think that it is important to consider the alternative caveat of different amounts of light entering the system. Changes in light level caused by pupil diameter variations can be quite large. Please also note that I do not mean the luminance transient associated with the target onset. I mean the luminance of the gray display. it is a source of light. if the pupil diameter changes, then the amount of light entering to the visually sensitive neurons also changes.

Comments on revised manuscript:

The authors have addressed my first primary comment. For the light comment, I'm still not sure they addressed it. At the very least, they should explicitly state the possibility that the amount of light entering from the gray background can matter greatly, and it is not resolved by simply changing the analysis interval to the baseline pre-stimulus epoch. I provide more clear details below:

In line 194 of the redlined version of the article (in the Introduction), the citation to Baumann et al., PNAS, 2023 is missing near the citation of Jagadisan and Gandhi, 2022. Besides replicating Jagadisan and Gandhi, 2022, this other study actually showed that the subspaces for the visual and motor epochs are orthogonal to each other

We thank the reviewer for this comment and apologize that the citation to Baumann et al., PNAS, 2023 was missing in the previous version of the manuscript. In addition to including this citation in the revised version, we have provided a much more comprehensive description of all three cited studies and clarified that, in addition to replicating the results of Jagadisan and Gandhi, Baumann et al., PNAS, 2023 showed that the subspaces for the visual and motor epochs are orthogonal to each other. The following lines have been added to the Introduction of the revised manuscript:

“A similar separation has been observed for visual and motor responses in the SC (Jagadisan and Gandhi, 2022; Ayar et al., 2023; Baumann et al., 2023). For example, Jagadisan and Gandhi (2022) used linear microelectrode arrays to investigate why early eye movements are not triggered when neuronal responses to a visual target, presented before a delayed saccade to that target, cross a threshold. They found that population activity in the SC was less stable during the visual epoch of a delayed saccade task, relative to the saccade epoch. Moreover, saccades could be evoked more easily by patterned microstimulation when the temporal structure of the microstimulation was stable across electrodes, providing a potential explanation for how downstream regions differentiate between visual and motor responses. Similar results were reported by Baumann et al. (2023) who found that the strength of SC motor responses during a saccade to a visual image depends on the features of that image (e.g., contrast, orientation). When dimensionality reduction was applied to the spiking responses of neuronal populations in the SC, the population trajectory during the initial visual response to the image was orthogonal to that during the motor response. These findings replicate the separation in temporal population structure reported by Jagadisan and Gandhi (2022) and support the results of Ayar et al. (2023). They found that, although not completely orthogonal, population activity in the SC is distinct for visual and motor responses during the same oculomotor task and across different tasks, which could further facilitate the decoding of signals related to sensation, action and context by downstream regions.” (Lines 110-127)

Line 683 (and around) of the redlined version of the article (in the Results): I'm very confused here. When I mentioned visual modulation by changed pupil diameter, I did not mean the transient changes associated with the brief onset of the cue in the memory-guided saccade task. I meant the gray background of the display itself. This is a strong source of light. If the pupil diameter changes across trials, then the amount of light entering the eye also changes from the gray background. Thus, visually-responsive neurons will have different amount of light driving them. This will also happen in the baseline interval containing only a fixation spot. The arguments made by the authors here do not address this point at all. So, please modify the text to explicitly state the possibility that the global luminance of the display (as filtered by the pupil diameter) alters the amount of light driving the visually-responsive neurons and could contribute to the higher effects seen in the more visual neurons.

We apologize that our analysis did not fully address the reviewer’s concern that the presence of fluctuations in visual neurons and their absence in motor neurons may have arisen indirectly due to changes in the amount of light entering the eye caused by changes in pupil size. As per the reviewer’s suggestion, we have now raised the possibility that visual neurons in the SC may have firing rates that are monotonically related to slow trends in overall luminance induced by pupil size changes, whereas motor neurons do not. Although we believe this to be an unlikely explanation, the paragraph from lines 374-398 has been modified to better describe this possibility, including the following text:

“Given that slow drift is found in traditionally defined visual areas (e.g., area V4) and in regions that show mixed selectivity for multiple task variables (e.g., PFC) (Cowley et al., 2020), it seems unlikely that slow drift is caused by luminance fluctuations alone and more likely that it reflects global changes in arousal. At the same time, these arousal-related fluctuations covary with changes in pupil size (Johnston et al., 2022a), which could modulate the amount of light entering the eye from the display. This might affect visual neurons but not motor neurons due to their lack of visual sensitivity. Because SC neurons exist on a continuum, with visual responses decreasing and motor responses increasing from the intermediate to deep layers (Massot et al., 2019; Heusser et al., 2022) and no clear categorical boundary for motor-only neurons, any readout strategy would still need to avoid corruption of the motor output by slow drift, even if it were caused by changes in the amount of light entering the eye.” (Lines 387-398)

The figures (everywhere, including the responses to reviewers) are very low resolution and all equations in methods are missing.

We thank the reviewer for bringing this to our attention. We believe this issue may have arisen during conversion of the manuscript file for review, as the figures were of sufficient quality and the equations visible in the version that appeared online (https://doi.org/10.7554/eLife.99278.2). In any case, we will ensure that high-resolution figures are submitted with the revised manuscript and apologize that they were low resolution in the previous version.

I'm very confused by Fig. 2 - supplement 2. Panel B shows a firing rate burst aligned to *microsaccade* onset. Does that mean you were in the foveal SC? i.e. how can neurons have a motor burst to the target of the memory-guided saccade and also for microsaccades? And which microsaccade directions caused such a burst? And what does it mean to compute the motor index and spike count for microsaccades in panel C? if you were in the proper SC location for the saccade target, then shouldn't you *not* get any microsaccade-related burst at all? This is very confusing to me and needs to be clarified

We agree that clarification is needed here and thank the reviewer for their comment. The eccentricity of the targets was set to match the endpoints of the evoked saccades, which for some sessions were relatively close to the fovea. The mean eccentricity of the targets across sessions was 4.52° (SD = 2.89°). These values are now reported in the Methods section of the revised manuscript (Line 637). For the neuron shown in Figure 2–figure supplement 2, the eccentricity of the targets was 3°. Previous research has shown that some SC neurons respond during microsaccades as well as slightly larger saccades (see Hafed & Krauzlis, 2012, J. Neurophysiol., Fig. 4B). This likely explains why the neuron shown in Figure 2–figure supplement 2, which had a receptive field at ~3° based on saccades evoked by microstimulation, also responded during microsaccades. We apologize that this was not explained in the previous version and agree that it could have been confusing for the reader. To address this, the legend for this supplementary figure has been edited in the revised version and now reads:

“(B) PSTH for an SC neuron that responded around the time of a microsaccade. Firing rates were computed in 1ms bins, averaged across trials and smoothed using a Gaussian function (σ = 5ms). Note that the targets were set to 3º in this session based on saccades evoked by microstimulation (see Methods). Previous research has shown that some SC neurons respond during microsaccades as well as to slightly larger saccades (Hafed and Krauzlis, 2012). This likely explains why this SC neuron, which had a RF at ~3º based on saccades evoked by microstimulation, also responded around the time of a microsaccade.” (Lines 1026-1031)
